# Complete Genome Sequences of Six *Chrysodeixis includens* Nucleopolyhedrovirus Isolates from Brazil and Guatemala

**DOI:** 10.1128/genomeA.01192-16

**Published:** 2016-12-08

**Authors:** Saluana R. Craveiro, Luis Arthur V. M. Santos, Roberto C. Togawa, Peter W. Inglis, Priscila Grynberg, Zilda Maria A. Ribeiro, Bergmann M. Ribeiro, Maria Elita B. Castro

**Affiliations:** a Embrapa Recursos Genéticos e Biotecnologia, Parque Estação Biológica, Brasília, Distrito Federal, Brazil; b Departamento de Biologia Celular, Universidade de Brasília—UnB, Brasília, Distrito Federal, Brazil; c Centro Universitário de Brasília—UniCEUB, Brasília, Distrito Federal, Brazil

## Abstract

The baculovirus, *Chrysodeixis* (formerly *Pseudoplusia*) *includens* nucleopolyhedrovirus (ChinNPV), is a new *Alphabaculovirus* pathogenic to *Chrysodeixis includens*. Here, we report the complete genome sequences of six ChinNPV isolates. The availability of these genome sequences will provide information on ChinNPV molecular genetics, promoting understanding of its pathogenicity, diversity, and evolution.

## GENOME ANNOUNCEMENT

*Chrysodeixis includens* nucleopolyhedrovirus isolates, obtained from *Chrysodeixis includens* larvae collected from cotton and soybean crops from Brazil and Guatemala exhibited genetic and phenotypic diversity in previous studies (1, 2). These viral isolates were initially named as *Pseudoplusia includens* single nucleopolyhedrovirus (PsinSNPV). However, the nomenclature of this new baculovirus species was reviewed and its name revised to *Chrysodeixis includens* nucleopolyhedrovirus (3).

Here, genomes of six isolates, namely, ChinNPV-IA, ChinNPV-IB, ChinNPV-IC, ChinNPV-ID, ChinNPV-IF, and ChinNPV-IG were sequenced using the Roche 454 GS FLX−Titanium platform. Genome assembly was performed using Newbler Assembler v2.8 (Roche), MIRA v4.0.2 (4), Celera Assembler v8.3 (5), Geneious v6.1.8 (6), and open reading frame (ORF) prediction was carried out with ORF Finder (NCBI) and Geneious v6.1.8.

The six isolates exhibited genome sizes ranging from 138,869 bp (ChinNPV-IB) to 140,859 bp (ChinNPV-IF), with an average G+C content of 39.2%. The isolates showed high nucleotide sequence identity with both ChinNPV-IC and ChinNPV-ID sharing 96.4% identity and ChinNPV-IF and ChinNPV-IG sharing 99.4% identity with the PsinSNPV-IE genome (7) (GenBank: KJ631622), representative isolate of the species.

Structural rearrangements such as large-scale inversions and translocations were absent. Genetic heterogeneity was observed among these seven virus isolates with the presence of indels (deletions or insertions), single nucleotide polymorphisms (SNPs), and small fragment insertions throughout the genome. The genomes of all the ChinNPV isolates exhibited two *bro* genes (*bro-a* and *bro-b*), similar to the PsinNPV-IE genome. However, an insert of approximately 1,700 bp was mapped between the *pif-3* and *sod29* genes which resulted in the identification of a third *bro* gene (*bro-c*) in the ChinNPV-IA, ChinNPV-IC, and ChinNPV-ID genomes.

Single nucleotide polymorphisms observed among the ChinNPV genomes may also significantly contribute to phenotypic diversity and different degrees of pathogenicity previously reported among the seven isolates herein analyzed (1).

The complete genome sequences presented here will be invaluable for further molecular genetic studies that could provide new insights into the virulence, insect-host interactions, and phenotypic diversity observed among ChinNPV isolates. In addition, this genomic data will contribute to advances in the understanding of genetic diversity and to functional studies of baculovirus genes.

### Accession number(s).

ChinNPV isolate genome sequences have been deposited in GenBank under the following accession numbers: KU669289 (for ChinNPV-IA), KU669290 (for ChinNPV-IB), KU669291 (for ChinNPV-IC), KU669292 (for ChinNPV-ID), KU669293 (for ChinNPV-IF), and KU669294 (for ChinNPV-IG).
